# Walking on the bright side: Associations between affect, depression, and gait

**DOI:** 10.1371/journal.pone.0260893

**Published:** 2021-12-02

**Authors:** Divya Kumar, Dario J. Villarreal, Alicia E. Meuret

**Affiliations:** 1 Department of Psychology, Southern Methodist University, Dallas, Texas, United States of America; 2 Department of Engineering, Southern Methodist University, Dallas, Texas, United States of America; University of Rochester, UNITED STATES

## Abstract

**Background:**

Psychomotor change is a core symptom of depression and one of the criteria in diagnosing depressive disorders. Research suggests depressed individuals demonstrate deviations in gait, or walking, compared to non-depressed controls. However, studies are sparse, often limited to older adults and observational gait assessment. It is also unclear if gait changes are due to dysregulation of affect, a core feature of depression. The current study addressed this gap by investigating the relation between positive and negative affect, depressive symptom severity, and gait in young adults.

**Methods:**

Using three-dimensional motion capture, gait parameters (velocity, stride length, and step time) were attained from 90 young adults during a task where they walked ten meters at their own pace overground in a laboratory for ten minutes. Self-report measures of mood and affect were collected.

**Results:**

On average, the study population reported high negative and low positive affect. Contrary to our hypotheses, hierarchical regressions demonstrated no significant associations between gait parameters and affective or depressive symptoms (*p*s>.05).

**Conclusions:**

Our findings do not support a relation between affective symptoms and gait parameters. The results may indicate age-dependent gait pathology or that other symptoms of depression may influence gait more strongly than affect. They may also reflect an observational bias of gait changes in depressed young adults, one that is unsupported by objective data. Replication is warranted to further examine whether affective symptomology is embodied via gait differences in young adults.

## Introduction

Psychomotor change is one of nine symptoms a person can endorse to be diagnosed with major depressive disorder (MDD) in *The Diagnostic and Statistical Manual for Mental Disorders– 5*^*th*^ *edition* (DSM-5; [1]). Motor differences in MDD can encompass psychomotor agitation, which refers to an increase in bodily movement, speech, or thoughts, or psychomotor retardation, a slowing of these components [2–4]. Despite being commonly reported [5], much of the relation between psychomotor changes and other symptoms of MDD remains understudied. Given that motor changes directly impact an individual’s overall level of functioning, as difficulties initiating and controlling movement can affect a variety of domains, a better understanding of how motor symptoms relate to specific symptomology is needed.

To date, the majority of studies examining psychomotor changes in MDD utilize self or clinician-report. Diagnostic measures often contain a single item to assess motor change and ratings are based on clinical judgment, often validated for those who score at a moderate-to-severe range of pathology, distinguishing healthy populations from the most ill. This norming does not account for mild-to-moderate symptoms that still cause distress and impairment. Beyond this, measures do not parse the cognitive (e.g., delayed thought processing) and motor components of psychomotor change [6]. While rated assessments of motor change are undoubtedly ecologic, the biobehavioral nature of motor symptoms requires cross-examination and validation with objective measures. In the absence, one may fall victim to well-known observational biases such as those discussed in schizophrenia [7] and anxiety [8].

Gait, or walking, is one aspect of psychomotor ability. Broadly, slower gait velocity has been linked to poor health outcomes, such as greater rates of physical illness and overall mortality [9, 10]. Despite its link to health, there is a dearth of information on the association between gait and depressive symptoms. A systematic review by Murri and colleagues [11] examining instrumental (or technology-based) analysis of balance and gait in depression found 21 studies on gait. The authors note a majority (*n* = 16, 76.19%) were on samples over the age of 65 years. Findings broadly suggest older individuals with MDD walk more slowly than controls [11]. Among those, most (*n* = 13) assessed gait based on observational timed tests via stopwatch. Dual-task paradigms, wherein participants also simultaneously complete cognitive tasks, further confound results. Only three studies in those over 65 used an electronic walkway to assess for gait [12–14]. Of these, two did not find significant differences between depressed individuals and healthy controls during single-task gait tests after controlling for covariates [13, 14]. These findings suggest as measurement of gait becomes more refined, its association with depression becomes less consistent, highlighting the need for more studies using objective measurement.

The remaining five studies reviewed by Murri et al. [11] employed instrumental analysis of gait in depressed individuals younger than 65 using more refined assessments such as electronic walkways [15, 16], insoles [17], photogrammetry [18], and three-dimensional motion capture [19]. These instruments allowed investigators to capture components of the gait cycle beyond speed, though findings were mixed. Differences were noted in velocity [15, 19], swing time [16, 17], and stride length [15, 18]. The range of average ages in these studies was from 40 to 48 years. An investigation by Michalak and colleagues [19] included a second study on a younger sample (*M*_age_ = 23.9 years); however, sad mood was induced. Sample sizes were small (when averaged, groups were around *N* = 12 for depressed individuals and *N* = 17 for healthy controls) and height and weight were rarely accounted for. In sum, methodological shortcomings muddy conclusions on gait differences; therefore, replication in younger populations remains warranted.

Importantly, across studies, gait has been examined in depressed versus non-depressed groups. In these studies, depression is demarcated by a categorical cutoff (either diagnostically or by a score on an inventory). Emerging literature suggests symptoms of psychological disorders should be represented dimensionally, rather than categorical cutoffs that may restrict range of symptoms [20]; therefore, measuring individual differences in depressive symptoms, rather than between-group ones, is important. Further, research has demonstrated the utility of examining specific symptoms of depression rather than the overall syndrome [21]. Given that there are roughly 1,000 unique symptom combinations that can all meet criteria for an MDD diagnosis, understanding the relations between specific symptoms may be a more fruitful avenue for investigation [21]. The DSM-5 structure of MDD emphasizes two core symptoms: depressed mood or anhedonia, which map onto dysregulations of the negative and positive valence system, respectively [1]. Examining how these two core symptoms of depression are related to psychomotor change will allow for an understanding for which individuals these concerns present.

In response, the first aim of the present study is to extend the literature on gait and core features of depression: positive and negative affect. Gait differences seen in those with depression mirror deviations seen in individuals with anxiety about falling [22–24], perhaps suggesting a unique role that affect broadly has on changing this symptom. Therefore, an understanding of how specific affective states relate to gait will provide stronger evidence that the mood-component of depression is what relates to psychomotor deviance. The second aim is to study this relation in a young adult sample. Emerging adulthood is close to the onset of affective disorders and is a crucial time point for increases in depressive symptom reports [25]. If gait is to be used to identify affective dysregulation, it seems prudent to examine if deviations are apparent at this timepoint. A final exploratory aim is to investigate the concordance between self-reported psychomotor change and differences in gait parameters using refined motion capture methodology. Given the reliance on self-report to indicate whether a depressed individual is experiencing psychomotor change, it is important to validate that reported psychomotor difficulties are associated with objectively measured differences.

Taken together, the present study is the first to examine gait variables in a young adult sample presenting with a range of positive affect, negative affect, and depressive symptoms (rather than selected based on the presence of a diagnosis). We hypothesized differences in gait between individuals higher in depressive severity and negative affect, and lower in positive affect, than those with presentations on the other end of the spectrum for symptoms and severity. Specifically, we hypothesize that those with symptoms frequently seen in a depressive diagnosis (greater depressive severity and negative affect, as well as lower in positive affect), will demonstrate reduced gait velocity, shorter stride length, and longer step times, in line with findings from the literature on gait differences in depressed individuals. Contrastingly, we expect those on the other end of the spectrum of these symptoms to demonstrate the opposite, in line with findings of gait patterns from healthy controls.

## Method

### Participants

The only prior investigation of gait in depression utilizing three-dimensional motion capture reported large effect sizes (*d*s > 0.8; [19]). Based on this, an *a priori* power analysis was conducted on G*Power 3.1.9.2 [26] for the primary analysis utilizing linear regression to examine relations between depressive symptoms, affective symptoms, and gait parameters, controlling for covariates (age, weight, height, and gender). With an alpha error probability of .05 and medium effect size, to attain 80% power a sample of 77 participants would be necessary.

90 individuals participated in the study between September and December 2019. Participants included undergraduate students receiving course credit for psychology classes (*n* = 78) and community volunteers (*n* = 12). The sample was predominately female (*n* = 64, 71.11%) with an average age of 20.58 years (*SD* = 2.74, range:18–29 years). The average body-mass index was in the normal range (*M* = 22.96, *SD* = 4.62, range: 16.72–38.13). Thirty-nine (43.33%) individuals in the study reported taking some regular form of medication, for symptoms relating to allergies, psychological difficulties, or contraception. See Table 1 for demographic information.

**Table 1:** Diagnostic and clinical characteristics of study participants.

*Demographic Information*	N = 90
Female, N (%)	64 (71.11)
Age, M (SD)	20.58 (2.74)
Race, N (%)	
White	49 (54.44)
Asian	20 (22.22)
African American	4 (4.44)
Non-White Latino	5 (5.56)
White Latino	2 (2.22)
Alaskan	1 (1.11)
Multiracial	6 (6.67)
Other	3 (3.33)
Height (cm), M (SD)	168.35 (10.01)
Weight (kg), M (SD)	66.01 (15.55)
BMI, M (SD)	22.96 (4.62)
Days of Week Exercise^1^, N (%)	
0 days	7 (7.87)
1–2 days	31 (34.44)
3–4 days	37 (41.11)
5–7 days	14 (15.56)
*Clinical Information*	
BDI-II, M (SD)	7.71 (7.95)
PANAS-P^2^, M (SD)	30.12 (6.99)
PANAS-N^2^, M (SD)	18.60 (6.43)
Psychomotor Retardation, N (%)	10 (11.11)
Psychomotor Agitation, N (%)	16 (17.78)

*Note*. BDI-II, Beck Depression Inventory–Second Edition; PANAS-P, Positive and Negative Affect Schedule–Positive Affect Subscale; PANAS-N, Positive and Negative Affect Schedule–Negative Affect Subscale; ^1^ = Missing one data point (*N* = 89); ^2^ = A PANAS-P score of below 32 is considered below the 50^th^ percentile in a non-clinical sample and a PANAS-N score of above 15 is considered above the 50^th^ percentile in a non-clinical sample [27].

Participants were excluded if they met criteria for current substance use disorder or suffered from psychotic symptoms, due to known relations with gait. Exclusion criteria were assessed using the Psychiatric Diagnostic Screening Questionnaire (PDSQ; [28]). Participants who had physical conditions that affected ability to complete a walking task or that impacted gait were also excluded. Only two individuals were ineligible due to substance use. Participants were requested to not consume caffeine or alcohol prior to the study session and to wear fitted pants or leggings and flat-soled shoes. The study was approved by the Southern Methodist University Institutional Review Board. All participants signed an informed consent and were debriefed following study completion.

### Self-report measures

#### Affective symptoms

The Positive and Negative Affect Schedule (PANAS; [29]) is a 20-item self-report measure assessing for levels of positive and negative affect. The PANAS consists of ten items for positive affect (PANAS-P; attentive, interested, alert, excited, enthusiastic, inspired, proud, determined, strong, active) and ten items for negative affect (PANAS-N; distressed, upset, hostile, irritable, scared, afraid, ashamed, guilty, nervous, jittery). Participants were asked to rate how much they felt each emotion that day on a scale from zero to five, with higher scores indicating greater intensity. A PANAS-P score of below 32 is considered below the 50^th^ percentile, while a PANAS-N score of above 15 is considered above the 50^th^ percentile in non-clinical samples [27]. The PANAS has an internal consistency of *α* = .89. Alpha for the PANAS-P for the current sample was .91 and for the PANAS-N was .86.

#### Depressive symptoms

The Beck Depression Inventory–Second Edition (BDI-II; [30]) is a 21-item self-report measure assessing for key symptoms of MDD such as negative cognitive distortions, sad mood, changes in appetite, etc., on a scale from zero (not present) to three (severe). The BDI has established ranges of mild, moderate, or severe depression. The scale also includes one item assessing for psychomotor agitation, but not retardation. The BDI-II has a test-retest reliability of .93 across one week and a coefficient alpha of .92 for depressed individuals in outpatient settings. The alpha of the current sample was .91, suggesting good internal consistency.

#### Psychiatric symptoms

The Psychiatric Diagnostic Screening Questionnaire (PDSQ; [28]) is a self-report questionnaire that aids with screening for common DSM-5 psychiatric disorders. The subscales relevant to screening for psychosis, alcohol use, and drug use were used to screen participants for inclusion in the study. Each subscale contains six "yes" or "no" items. The PDSQ has demonstrated adequate reliability and validity in patient samples, with test-retest reliability coefficients greater than .80 for each of the subscales used in the study.

#### Psychomotor retardation or agitation

Participants were also asked whether they had experienced psychomotor retardation (*"In the past two weeks*, *have you experienced symptoms of slowed thoughts or slower movements in your body*, *like you were moving through molasses*?*")* or psychomotor agitation (*"In the past two weeks have you had difficulty sitting still or felt much more agitated/restless*?*")*. These items were detailed on a demographic form (see below).

#### Demographic information and control variables

Participants completed a questionnaire on their current levels of physical health, age, and gender. Participant height and weight were collected during the laboratory session by study staff, based on which body-mass-index was calculated. These were included in the gait assessment system and statistical analyses as control variables. Other control variables (gender and age) were collected from the participant demographics form.

### Gait assessment

**Fig 1:**
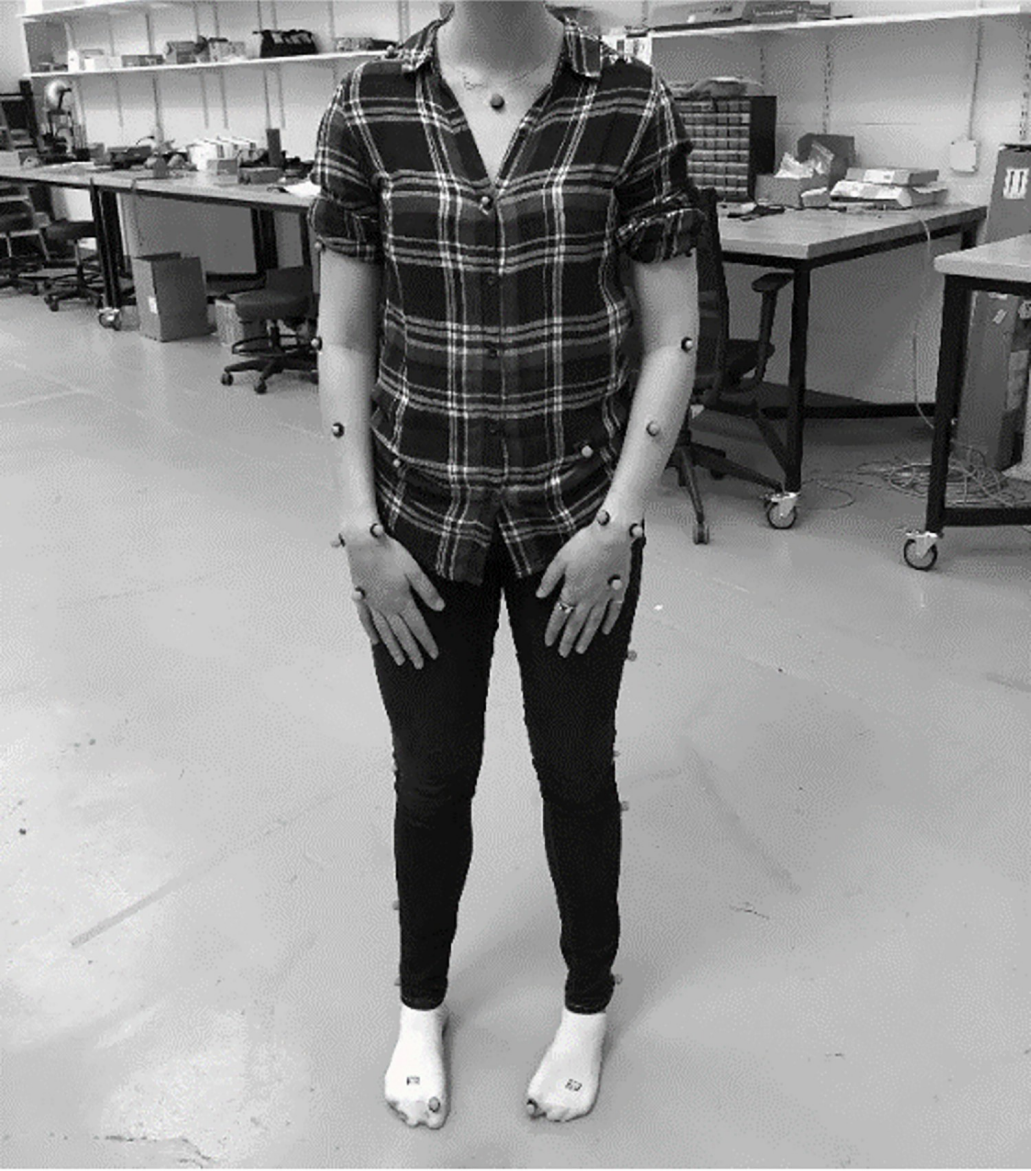
Depiction of subject with reflective markers of the Vicon system placed on lower and upper body. *Note*. Reflective markers from the lower body were utilized for the current investigation.

Gait parameters were captured using the Vicon Nexus motion analysis system (Vicon, UK). The Vicon system has been validated and is considered a gold-standard for motion and clinical gait analysis [31, 32]. It contains 16 cameras to capture a full range of motion to a precise temporal resolution (a sampling frequency of 1000 Hz). It is able to utilize reflective markers placed on participants extremities to produce coordinates of individual movements based on body position relative to X, Y, and Z-axes. Seventeen markers were placed on participants lower bodies. See Fig 1 for display of participant set-up with reflective markers. Static calibration was attained via the participant standing at a researcher-set point of origin. Based on an internal program, the system is able to calculate certain gait parameters, among which the key variables of interest for the current study were collected. An assessor is required to view recorded data of a participants walking and denote timepoints where there was a heel strike (when the participant’s heel touched the ground) and a toe off (when the participant’s toe left the ground). Heel strike and toe offs for each participant were denoted by two research assistants for agreement. Following this, the Dynamic Plug-in-Gait pipeline in the system is able to automatically detect gait cycle events and provide an output in CSV format, which we utilized for our current study.

### Data selection for gait parameters

Though the system’s pipeline for calculating gait parameters provides a variety of gait outputs, the present investigation focused on three lower-body parameters: average velocity, step time, and stride length. This allowed us to compare our findings to prior studies while maintaining power. While there are a number of gait outcomes that have been assessed in the literature, we chose these three for two main reasons. First, studies assessing gait changes in adults under the age of 65 with depression have found differences in these parameters [11]. In particular, much of the literature has noted a significant association between depression and gait velocity, with depressed individuals exhibiting slower gait [9, 11, 12, 15]. Additionally, stride length has been noted as significantly differing between depressed individuals and healthy controls [12, 15, 18]. Notably, both of these gait parameters remained significantly associated with depressive status after controlling for other covariates that might impact walking [12]; given that our investigation aimed to likewise control for related factors, we chose to investigate both of these parameters. Finally, step time has been noted as a feature of gait that can be analyzed, however few studies with younger adults examine this [11]. Given that it is a discrete gait outcome and because it may be that step time relates to overall reduced gait velocity, we chose to include this exploratory aspect of gait as well. Second, and in line with this former statement, we aimed to pick discrete gait variables that had the potential to be measured in clinical settings without the use of refined gait analysis technology (i.e., those that could be visible by an observer). If we noted significant differences in these parameters in our study, clinicians could feasibly measure or observe them for diagnostic purposes.

Participants were instructed to conduct a standard 10-minute uninterrupted walking test (see below). Gait cycle parameters on the Vicon system were calculated using only the last two minutes of walking data via the Vicon Nexus Dynamic Plug-in-Gait pipeline. Doing so controls for potential confounds that could influence a participant’s gait (e.g., novelty of laboratory environment). We theorized that an 8-minute habituation period would result in a more natural gait. This decision mirrors those made in prior examinations of gait to allow participants to habituate to the lab environment [19].

*Gait velocity* was operationalized as stride length divided by stride time, referring to the velocity at which a participant is walking in the X-direction, measured in meters per second.

*Stride length* was defined as the distance between the foot marker at foot’s first contact and the same marker at the same foot’s subsequent step, measured in meters.

*Step time* was measured as the duration that each foot was on the ground before being lifted into swing phase, measured in seconds.

### Procedures

Individuals who met inclusion criteria were informed that they would be taking part in a research experiment examining correlates of physical and psychological well-being. Students were informed that the study was a collaboration between the psychology and engineering departments and that data collection would take place in the NeuroMechatronics Laboratory.

**Fig 2:**
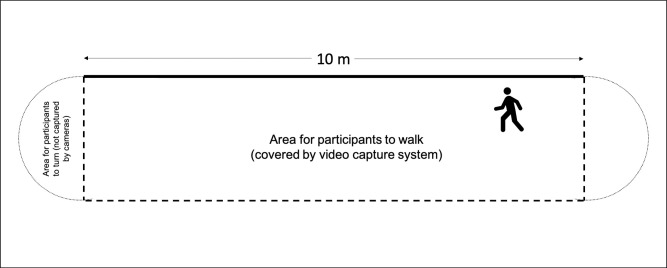
Schematic diagram of walking task.

Participants partook in a single laboratory session where they first completed a series of self-report questionnaires. Following this, physical health data was collected by study staff. Participants’ height, weight, and other relevant parameters (leg length, knee width, and ankle width) were measured using calibrated scales. Reflective markers were placed on participants’ lower bodies to be identified by the motion-capture system. Following system calibration, participants were asked to complete the gait task. This involved walking along a 10-meter pathway, denoted by tape on the floor (see Fig 2). Participants were told to walk alongside the tape as naturally as possible; therefore, they were not instructed they needed to walk in a completely straight line. The width of space participants had to walk from the denoted tape line to the wall of the laboratory was roughly three meters. When reaching the end of the ten meters, participants were told to walk past the end of the taped section of the floor and turn around without coming to an abrupt stop and continue walking again. Therefore, turns and any acceleration of gait was not captured by the Vicon camera and only segments where participants were walking straight ahead were analyzed. Upon completion of ten minutes of walking, markers were removed and participants were debriefed.

### Statistical analysis

We computed the descriptive statistics for all variables of interest. Correlations between independent and dependent variables were attained to assure relations were in the expected direction. To account for non-normal distributions and substantial positive skew, the BDI, stride length, and step time variables were log10 transformed. Hierarchical linear regressions were conducted to assess the predictive power of affective symptoms on gait parameters. The assumptions necessary to perform regression (normality of residuals, no multicollinearity of the independent variables) were checked. Three total models were run with each gait variable as a dependent variable. Covariates that are expected to relate to an individual’s gait (height, weight, age, and gender) were included as a first step in each model. As BMI is a product of an individual’s height and weight, this was not additionally added as a covariate. The predictors (PANAS-N, PANAS-P, and BDI) were added in the second step to note relation above covariates. Statistical analyses were conducted in IBM SPSS version 25. Bonferroni corrections were utilized to control for multiple tests.

## Results

### Descriptives and preliminary analyses

On average, PANAS-P scores (*M* = 30.12, *SD* = 6.99, range: 15–45) and PANAS-N scores (*M* = 18.60, *SD* = 6.43, range: 10–39) were at the 41st and 78th percentiles for positive and negative affect respectively as compared to the 50th percentile population average [27], suggesting lower levels of positive affect and higher levels of negative affect than is seen in a normative population. The sample had a mean BDI score of 7.71 (*SD* = 7.95, range 0–34). Seventeen (18.89%) individuals scored above 18, suggesting moderate-to-severe levels of depression, while the rest *(n* = 73, 81.11%) were in the mild range. Averages for gait outcome variables were: 0.93 meters per second (*SD* = 0.26, range: .22–1.50) for velocity, 0.80 seconds (*SD* = 1.05, range: .33–9.65) for step time, and 1.15 meters (*SD =* 0.35, range: .25–3.23) for stride length.

As expected, bivariate correlations were significant between the BDI and PANAS-N (*r* = .57, *p* < .001) and the BDI and PANAS-P (*r* = -.55, *p* < .001). The relation between both PANAS scales trended towards significance (*r* = -.21, *p* = .05). Stride length was significantly related to velocity (*r* = .65, *p* < .001) and step time (*r* = .54, *p* < .001). Correlations between variables in the analyses can be seen in Table 2. Few participants reported experiencing psychomotor change: 10 (11.11%) reported slowing and 16 (17.78%) reported psychomotor agitation.

**Table 2:** Bivariate correlation table for study variables.

	**1**	**2**	**3**	**4**	**5**	**6**	**7**	**8**	**9**
1. Age	**-**	-.02	-.10	-.07	.13	-.07	-.10	-.03	-.06
2. Height		**-**	.62**	-.30**	.06	-.12	.24*	.02	.25*
3. Weight			**-**	-.19	.03	-.06	.16	-.08	.06
4. Depressive Symptoms				**-**	-.55**	.57**	.04	.18	.18
5. Positive Affect					**-**	-.21	-.17	-.17	-.23*
6. Negative Affect						**-**	.04	-.04	.03
7. Gait Velocity							**-**	-.12	.65**
8. Step Time								**-**	.54**
9. Stride Length									**-**

*Note*.

* p < .05

** p < .01.

### Gait parameters, depressive symptoms, and affective levels

#### Gait velocity

**Fig 3:**
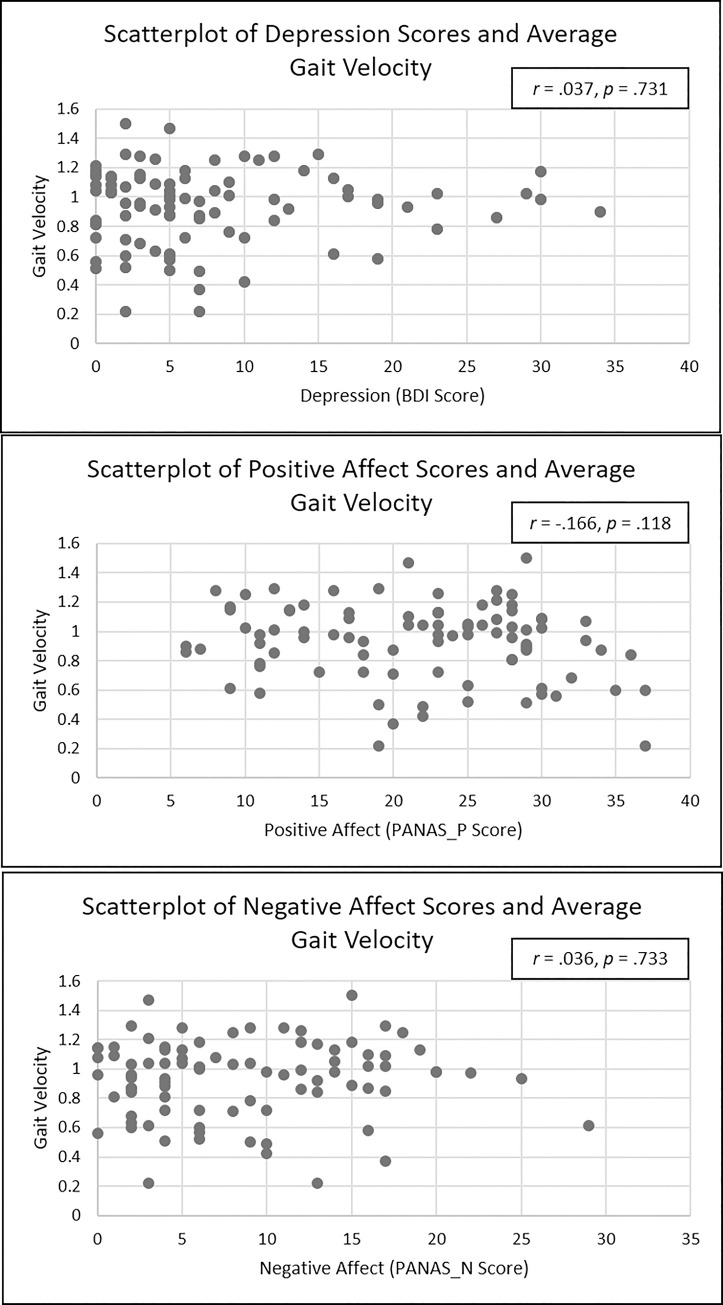
Scatterplots depicting relations between average gait velocity and depressive severity (BDI), positive affect (PANAS-P), and negative affect (PANAS-N).

Contrary to our expectations, results demonstrated no significant association between any of the predictor variables (PANAS-N, PANAS-P, and BDI) and gait velocity. See Table 3 for regression table. No significant associations were seen between severity of PANAS-N (*b* = 0.00, *SE* = .01, *p* = .59, 95% CI [-.01, .01], *sr*^2^ = .00) or PANAS-P (*b* = -0.01, *SE* = .01, *p* = .17, 95% CI [= -.02, .00], *sr*^2^ = .02) and gait velocity. Likewise, the relation between BDI score and gait velocity was not significant (*b* = -0.05, *SE* = .10, *p* = .64, 95% CI [-.25, .15], *sr*^2^ = .00). None of the covariates (height, weight, age, and gender) were significant in predicting gait velocity. Fig 3 depicts scatterplots of the relation between each independent variable and gait velocity.

**Table 3:** Regression results for gait velocity.

	Step 1				Step 2			
	*b*	*SE*	95% CI	*p*	*b*	*SE*	95% CI	*p*
Intercept	.53	.66	[-.78, 1.83]	.43	.59	.74	[-.88, 2.06]	.43
Age	-.01	.01	[-.03, .01]	.26	-.01	.01	[-.03, .01]	.41
Height	.01	.01	[-.01, .03]	.23	.01	.01	[-.01, .03]	.24
Weight	.00	.00	[-.00, .00]	.71	.00	.00	[-.00, .00]	.76
Gender	-.10	.08	[-.25, .06]	.24	-.08	.08	[-.25, .09]	.34
BDI					-.05	.10	[-.25, .15]	.64
PANAS_P					-.01	.01	[-.02, .00]	.17
PANAS_N					.00	.01	[-.01, .01]	.59
Δ*R*^2^	.08			.11	.03			.52

#### Average stride length

**Fig 4:**
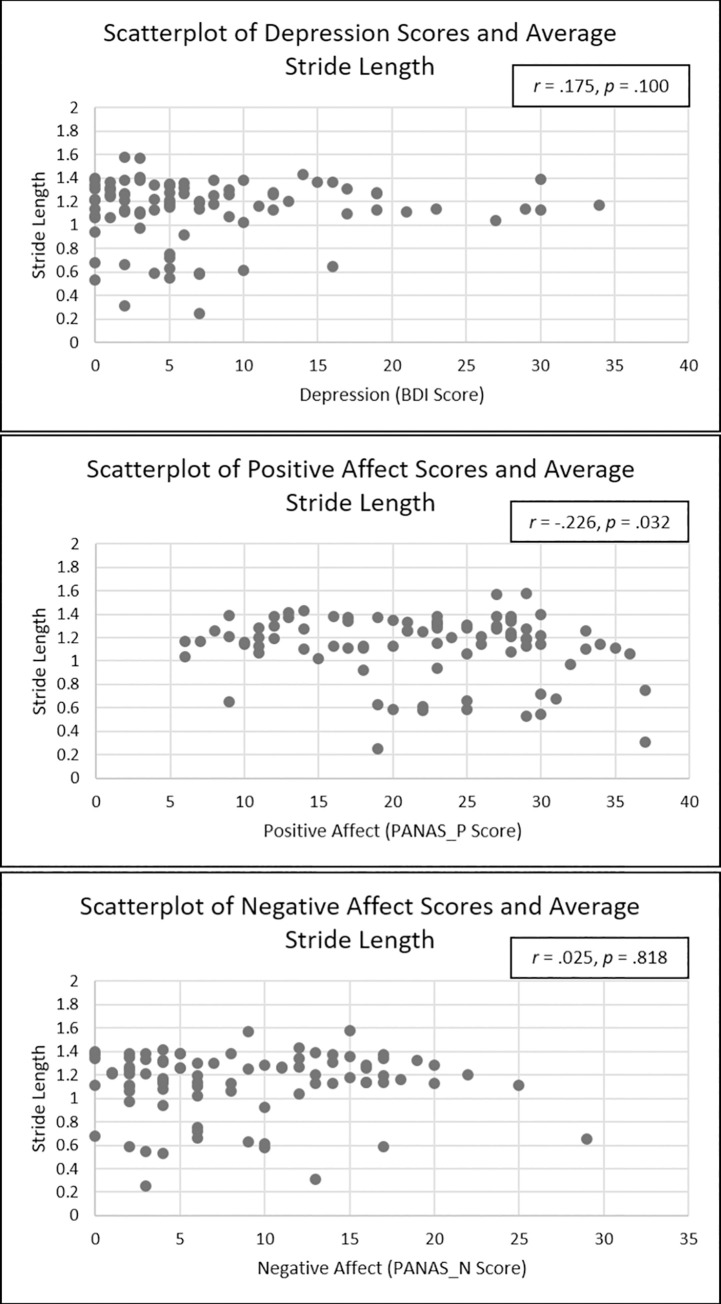
Scatterplots depicting relations between average stride length and depressive severity (BDI), positive affect (PANAS-P), and negative affect (PANAS-N).

Similarly, individuals with higher levels of PANAS-N (*b* = -0.00, *SE* = .00, *p* = .57, 95% CI [-.00, .00], *sr*^2^ = .00) and PANAS-P (*b* ***=*** -0.00, *SE* = .00, *p* = .14, 95% CI [-.00, .00], *sr*^2^ = .02) did not demonstrate significantly different stride lengths than those with lower levels. Those with higher BDI scores (*b* = 0.03, *SE* = .03, *p* = .34, 95% CI [-.03, .08], *sr*^2^ = .01) also had a non-significant relation with stride length. Height was the only significant covariate in the analyses after controlling for the independent variables and other covariates. Taller individuals demonstrated greater stride length (*b* = 0.01, *SE* = .00, *p* = .01, 95% CI [.00, .01], *sr*^2^ = .07). After using Bonferroni correction for multiple tests (three models, one for each gait parameter), this association retained significance. See Fig 4 for scatterplots of the relation between the predictor variables and stride length and Table 4 for regression results.

**Table 4:** Regression results for stride length.

	Step 1				Step 2			
	*b*	*SE*	95% CI	*p*	*b*	*SE*	95% CI	*p*
Intercept	.06	.18	[-.29, .42]	.72	-.03	.19	[-.41, .36]	.90
Age	-.00	.00	[-.01, .00]	.38	-.00	.00	[-.01, .00]	.58
Height	.01	.00	[.00, .01]	.04	.01	.00	[.00, .01]	.01
Weight	.00	.00	[-.00, .00]	.38	.00	.00	[-.00, .00]	.48
Gender	-.00	.02	[-.05, .04]	.84	.01	.02	[-.03, .05]	.66
BDI					.03	.03	[-.03, .08]	.34
PANAS_P					-.00	.00	[-.00, .00]	.14
PANAS_N					-.00	.00	[-.00, .00]	.57
Δ*R*^2^	.07			.16	.06			.12

#### Average step time

**Fig 5:**
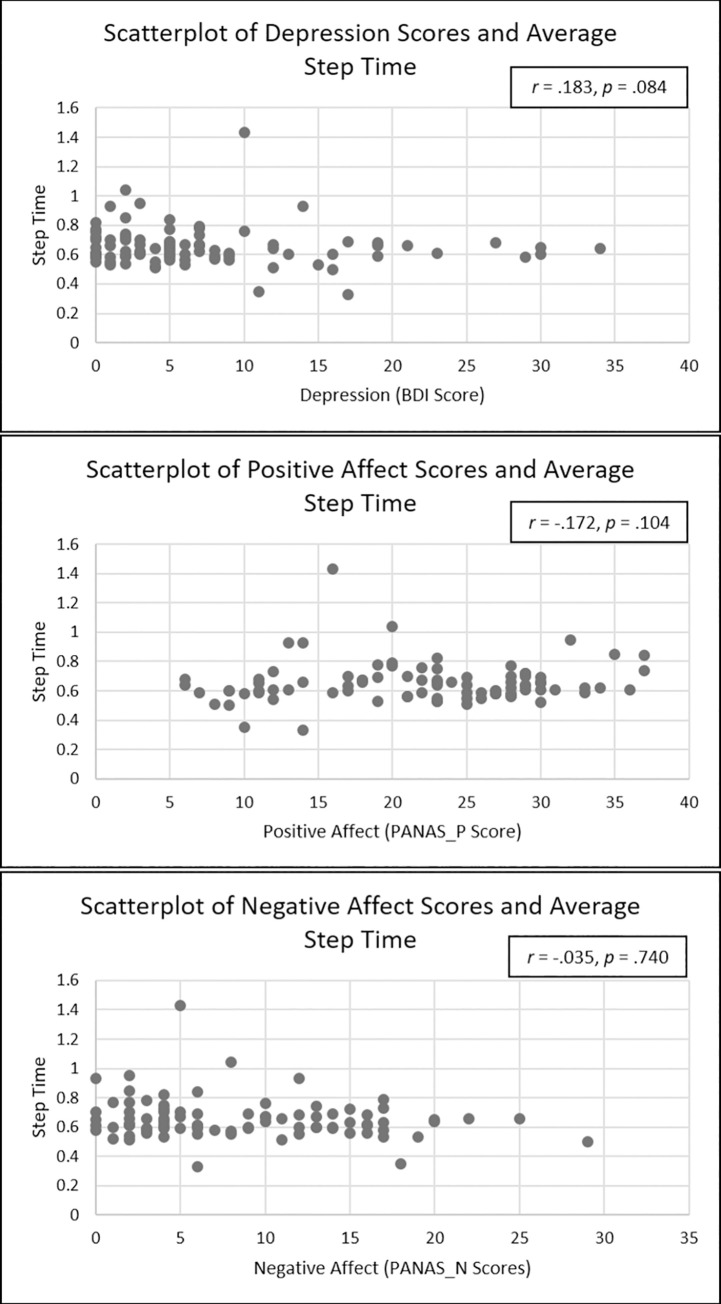
Scatterplots depicting relations between average step time and depressive severity (BDI), positive affect (PANAS-P), and negative affect (PANAS-N).

See Table 5 for regression results. Individuals with greater BDI scores did not have significantly different step time than those with lower ones (*b* = 0.08, *SE* = .04, *p* = .06, 95% CI [= -.00, .17], *sr*^2^ = .04). Similarly, after correcting for multiple tests, negative affect (PANAS-N) did not significantly predict step time (*b* = -0.01, *SE* = .00, *p* = .05, 95% CI [-.01, .00], *sr*^2^ = .04). Finally, those with lower levels of positive affect (PANAS-P) did not significantly differ from those with higher levels in their step time (*b* = -0.00, *SE* = .00, *p* = .60, 95% CI [-.01, .00], *sr*^2^ = .00). None of the covariates were significant in predicting average step time. See Fig 5 for scatterplots of the relation between BDI, both PANAS subscales, and step time.

**Table 5:** Regression results for step time.

	Step 1				Step 2			
	*b*	*SE*	95% CI	*p*	*b*	*SE*	95% CI	*p*
Intercept	.12	.28	[-.44, .68]	.67	-.07	.31	[-.68, .55]	.83
Age	-.00	.00	[-.01, .01]	.61	-.00	.00	[-.01, .01]	.68
Height	.00	.00	[-.01, .01]	.39	.01	.00	[-.01, .02]	.12
Weight	-.00	.00	[-.00, .00]	.26	.00	.00	[-.00, .00]	.35
Gender	-.00	.03	[-.07, .07]	.95	.02	.04	[-.05, .09]	.51
BDI					.08	.04	[-.00, .17]	.06
PANAS_P					-.00	.00	[-.01, .00]	.60
PANAS_N					-.01	.00	[-.01, .00]	.05
Δ*R*^2^	.02			.80	.07			.09

### Post-hoc, exploratory analyses

#### Dichotomized sample

We dichotomized the sample along depressive severity to compare our results to those seen in all prior instrumental studies examining gait in severely depressed individuals (for review of prior studies, reference Murri et al. [11]). Given standard BDI cut-off scores, those with a score of 19 or greater (*n* = 17) (moderate-to-severe depression) were compared to those with lower scores (*n* = 73) (mild-to-no depression). Notably, as our depressed versus not-depressed groups were largely unbalanced, such findings should be interpreted with caution; however, we included this analysis to determine whether our null findings were due to the differences in statistical analysis in our study versus those prior. No significant difference in the between the two groups was detected. That is, individuals with moderate-to-severe depression did not differ in predicting velocity (*t*(88) = -.67, *p* = .53, 95% CI [-.19, .10], *d* = .19) or step time (*t*(88) = -1.02, *p* = .31, 95% CI [-.09, .03], *d =* .20) to individuals who were only mildly or not depressed. Those in the severely depressed group had greater stride length (*t*(88) = -1.86, *p* = .07, 95% CI [-.07, .00], *d =* .47), though this was not significant.

#### Psychomotor agitation versus retardation

We examined whether those who endorsed psychomotor agitation or retardation differed on gait parameters. When comparing those who reported psychomotor retardation (*n* = 10) versus those who did not (*n* = 80), the results of independent samples t-tests demonstrated there were no significant mean differences on any gait parameters (*p*s>.05, *d*s = .05 - .14). Similarly, when comparing those who reported psychomotor agitation (*n* = 16) to those who did not (*n* = 74), there were again no significant mean differences across parameters (*p*s>.05, *d*s = .01 - .19). Taken together, findings suggest little concordance between self-reported changes in psychomotor functioning and measured differences in gait.

## Discussion

This study is the first to our knowledge to examine relations between gait parameters and affective and depressive symptoms dimensionally using state-of-the-art gait assessment technology. Prior studies did not specify relations between specific affective symptoms of depression and gait. Our results demonstrate that as positive and negative affect, as well as depressive, severity increases, there are not significant predictive relations with gait parameters. Our study provides several contributions that may explain the discrepancy from prior literature and expand our understanding of this relation in young adults.

The primary distinctions between our investigation and priors were methodological. In addition to adopting a symptom-based approach, we also controlled for third variables that many former studies did not. Given gait is a multidimensional construct affected by various factors and there is evidence that height, age, and weight contribute to differences in gait in healthy adults [33, 34], we statistically controlled for these. In support, we noted significant correlations between height and gait velocity and stride. In prior studies comparing groups, physical features may have impacted gait. For instance, Michalak et al. [19] noted significant weight differences between groups but did not control for this. Further, we assessed gait using a state-of-the-art three-dimensional motion capture system. This allows for a full range of motion with minimal obtrusion. Many prior studies in adults utilized measurement which may inhibit natural gait [11]. By contrast, three-dimensional motion capture may more closely mirror everyday walking and increase generalizability. Allowing participants to acclimate to the task and setting also may have aided with external validity. Notably, other studies have found differences in other gait parameters, such as variability and upper-body postural changes, which we did not include in our investigation. While we chose the three gait variables based on what seemed most feasible to measure in clinical practice, the differences we noted in our findings may be due to the fact that we did not measure these other outcomes.

We also utilized a large sample with varying degrees of affective symptoms. Prior samples of adults compare healthy controls to depressed individuals, with many recruiting from inpatient units (e.g., [11]). Thus, findings do not generalize to mild-to-moderately symptomatic individuals. In contrast, individuals in our study ranged from low to clinical levels of negative and positive affect, and no associations were seen with gait. The examination of these core mood symptoms of depression provide information on how much the association between gait and depression may be due to mood. Differences in our results between the predictive relation of the depressive symptoms and affect suggest that prior differences between groups, using categorical diagnosis, might be due to other symptoms of MDD.

To our knowledge, no other study has used instrumental analysis to examine gait and affective symptoms in young adults. As emerging adulthood is a time nearest to the onset of depressive disorders [25], examining gait in this group is warranted. Changes in gait may be a result of age as opposed to depression. In support, Patience et al. [35] detailed a connection between executive functioning, depressive symptoms, and slowed gait specific to later-life depression. This finding is consistent with aging studies that suggest the decline in executive function systems may serve as a pathway to decreased gait velocity in older adults [36, 37]. Such cognitive features could be relevant for the initiation and modification of gait. The conjunction of cognitive and health changes in occurrence with depression may manifest in gait differences in older adults [35]. Therefore, it is plausible to assume that gait differences are less relevant to earlier episodes of depression. Additionally, gait changes are likely confounded by comorbidity with physical health disorders [10, 38], the latter of which are less likely to be present in younger samples. In support, the majority of our study participants were physically active. Prior technology-based gait studies in depression did not report on participants’ fitness level, making it difficult to compare the samples. However, it may be safe to assume that individuals who are inpatient or severely depressed are less active than controls. Future studies should continue to investigate whether psychomotor changes vary between different developmental periods.

In line with this, it may be a conjunction of affect with these other symptoms that relate to gait changes. For instance, increases in negative affect within depression may be due to associated features of the disorder, such as rumination [39, 40]. Individuals who are engaged with ruminative thoughts may experience increased cognitive load, which distract attention away from their movements, or gait patterns. In support, dual-task studies of gait note differences when individuals are engaging in cognitive tasks than when they are simply asked to walk [16], noting that preserving cognitive function may aid with stability. It may be the case that individuals who are depressed and engaging with ruminative thoughts therefore exert less cognitive control over features of gait. In particular, as individuals age, this balance may be more difficult to maintain, perhaps contributing to the age-related differences seen in gait for younger versus older adults [22, 41–43]. In particular, older adults demonstrate different gait from younger ones, as evidenced by changes in velocity and variability [42, 43]. Therefore, differences seen in older samples with depression as compared to younger ones, such as ours, may be due to these age-related factors rather than affective symptoms. Therefore, future investigations should not only examine the conjunction of cognitive features of depression with affect in order to note how these combined symptoms may contribute to overall changes seen in gait for depressed individuals, but also continue to study whether there are age-related differences. Likewise, it may be the case that changes in positive and negative affect contribute to other depressive symptoms–such as changes in sleep, reduced appetite, etc.–that in turn affect aspects of gait. Investigation and inclusion of moderating variables between affective symptoms of depression and gait outcomes warrant further study.

In consideration of current clinical practice, the findings suggest young adults are less likely to demonstrate impaired or slowed gait due to affect dysregulation. Therefore, gait may not be a helpful clinical marker for onset or diagnosis of affective disorders in younger individuals. Additionally, self-report ratings may not align with an objective evaluation of gait. In our study, individuals who reported psychomotor change did not demonstrate significantly different gait from those who did not. These results warrant replication, however, as gait assessment in younger adults presenting with affective dysregulation is in its infancy. Further, given that gait is a single component of psychomotor disturbance, clinicians assessing psychomotor change via diagnostic interviews should parse what aspect of motor change an individual is experiencing. Assessing for reaction time, response to motor tasks, latency of speech and may be more relevant in this sample.

While this study provides a greater understanding of psychomotor change in young adults with affective and depressive symptoms, there are limitations. First, while our results suggest no significant association between gait and the core symptoms of depression (dysregulated affect), further studies on influencing factors such as cognitive function and sleep changes (i.e. insomnia) are warranted. Second, future studies should include assessment of upper-body postural changes. There are a number of gait variables that have been examined in prior investigations of depression and gait, including parameters such as gait variability [16, 17]. While we did not include these outcomes in our examination in order to maximize power and due to their greater difficulty to measure in clinical settings, we recognize that the gait variables we chose may limit generalization to overall gait. Therefore, replication of this study using these other metrics in a study that is powered to detect effects is warranted. Third, the assessment may have altered participants’ natural gait even though individuals were unaware of the study aims. Fourth, there were some limitations to our assessment of gait. We did not collect data on the reliability between the two coders for heel off and toe strikes for each participant’s gait data. Finally, though the average positive and negative affect in our sample was respectively higher and lower than the population average, depression severity was mild. Replication of these results in young adults with greater depressive severity is warranted.

In conclusion, our findings suggest gait may not be an indicator of the affective symptoms of depression in young adults. Further research replicating these findings and investigating changes across time, within-individuals, is needed. Additionally, identification of what components of motor movement may be disrupted in young adult depression should continue to be explored in future investigations.
